# Genetic variations in *PRKAA1* predict the risk and progression of gastric Cancer

**DOI:** 10.1186/s12885-018-4818-3

**Published:** 2018-09-25

**Authors:** Minbin Chen, Baohu Jiang, Bangshun He, Min Tang, Ping Wang, Li Chen, Jianwei Lu, Peihua Lu

**Affiliations:** 1 Department of Radiotherapy & Oncology, Kunshan First People’s Hospital Affiliated to Jiangsu University, Kunshan, Jiangsu Province China; 2 Department of Critical Care Medicine, The affiliated Yixing Hospital of Jiangsu University, Yixing, 214200 Jiangsu Province China; 3 General Clinical Research center, Nanjing First Hospital, Nanjing Medical University, Nanjing, 220006 Jiangsu Province China; 4 Departments of Medical biology, Wannan Medical College, Wuhu, Anhui Province China; 5 Department of Gastroenterology, Xuzhou Hospital of Traditional Chinese Medicine Affiliated to Nanjing University of Chinese Medicine, Xuzhou, China; 6 Departments of Medical Oncology, Jiangsu Cancer Hospital Affiliated to Nanjing Medical University, Jiangsu Province Institute of Cancer, Nanjing, Jiangsu Province China; 7 Department of Medical Oncology, Wuxi People’s Hospital of Nanjing Medical University, No. 299, Qingyang Road, Wuxi, 214023 Jiangsu Province China

**Keywords:** Gastric cancer, Polymorphism, *PRKAA1*, Prognosis

## Abstract

**Background:**

*PRKAA1* encodes α-subunit of 5-AMP-activated protein kinase (AMPK), which has been implicated in the pathogenesis of carcinoma of the stomach. Previous works have suggested that polymorphisms in the *PRKAA1* may be associated with the risk of non-cardiac gastric cancer (NCGC), but whether *PRKAA1* polymorphisms are related to clinical pathologic characteristics of gastric cancer and its clinical outcome is largely unknown.

**Methods:**

We carried out a case-control study including a total of 481 gastric cancer patients and 490 healthy controls. The genotypes of enrolled polymorphisms were identified with Sequenom MassARRAY platform.

**Results:**

This study showed that rs10074991 GG genotype (adjusted OR = 1.44, 95%CI:0.99–2.09, *p* = 0.056) has a borderline significantly increased risk for gastric cancer, which was consistent with the result of additive model (adjusted OR = 1.21, 95%CI:1.01–1.46, *p* = 0.042). In similar, an increased risk of gastric cancer was also observed for rs13361707 TC genotype (adjusted OR = 1.47, 95%CI: 1.01–2.14, *p* = 0.043; additive model: adjusted OR = 1.22, 95%CI: 1.02–1.47, *p* = 0.033). Furthermore, the rs154268 and rs461404 were also found associated with increased gastric cancer risk, which may be influenced by age, tumor type and differentiation, and tumor stage. Haplotype analysis indicated A-G-C-T-C-G haplotype (rs6882903, rs10074991, rs13361707, rs3805490, rs154268 and rs461404) is associated with increased risk of gastric cancer (OR = 1.29, 95%CI: 1.02–1.62, *p* = 0.035). The univariate analysis for overall survival (OS) revealed that both of rs10074991 and rs13361707 variants are associated with poor OS in patients with NCGC.

**Conclusion:**

This case-control study provided the evidence thatrs13361707CC, rs10074991GG, rs461404GG, and rs154268CC are associated with increased gastric cancer risk, especially for NCGC, and that patients with rs10074991 G or rs13361707 C allele have a poor OS.

**Electronic supplementary material:**

The online version of this article (10.1186/s12885-018-4818-3) contains supplementary material, which is available to authorized users.

## Background

Gastric cancer (GC) is one of the most common cancers worldwide and remains the leading cause of cancer related death [1]. The incidence of this disease varies with the geographical region and patient ethnicity. About 70% cases in the world were reported from developing countries, and Eastern Asian countries have the highest GC incidence and mortality [1, 2]. Although mechanism of gastric carcinogenesis is still not fully understood, environmental factors, such as high intake of salt, tobacco smoking, and particularly *Helicobacter pylori*(*H. pylori*) infection have been regarded as the risk factors for the disease [3].Genetic factors have also been found to contribute to the risk of GC, with the first-degree relatives of the GC patients tending to have about 1.3 to 3.0 fold higher relative risk for GC than those without relatives with GC [4].

To date, genetic variations have widely been shown to be associated with GC risk [5], with particular importance on the polymorphisms involved in the signal transduction pathways [6, 7]. The 5-AMP-activated protein kinase (AMPK) pathway has been implicated in a series of tumors including GC. This is a heterotrimeric protein that consists of an α-catalytic subunit and 2 regulatory subunits (β and γ), and the α-subunit is encoded either by *PRKAA1* or *PRKAA2* gene. Previous genome-wide association study (GWAS) identified the *PRKAA1* polymorphism rs13361707 as a risk factor for non-cardiac GC (NCGC) in a Chinese population [8]; however, these results were not successfully duplicated [9], which may be due to the different characteristics of enrolled participants, population stratification, and clinical pathologic characteristics of GC. It is also not known whether the polymorphisms in the *PRKAA1* gene are related to clinical pathological characteristics of GC and clinical outcome of the patients. We carried out this case-control study on a Chinese population to investigate the susceptibility of six polymorphisms in the *PRKAA1* gene (see Additional file 1.) to the risk of GC and their associations with the clinical pathological characteristics, and evaluated the predictive value of these polymorphisms to the clinical outcome of GC patients.

## Methods

### Study subjects

A total of 481 GC patients, and age- and gender-matched 490 healthy individuals were enrolled in this study. The patients were histologically diagnosed as GC from Nanjing First Hospital, Nanjing Medical University, and the healthy controls were individuals who came to the hospital for routine physical examinations and were confirmed be healthy. All the participants were the heritably unrelated ethnic Han Chinese from the same geographic region of Nanjing City, Jiangsu, China. The whole blood of all enrolled participants were collected before operation and then stored at − 80 °C before genotyping. The clinical features of patients, including tumor size, distant metastasis, and depth of invasion, were collected from the patients’ medical records provided by Department of pathology, and the tumor TNM stages were examined and evaluated using the TNM classification according to American Joint Commission for Cancer Staging in 2002, sixth edition. The clinical outcomes of patients were found through on-site interview, direct calling, or medical chart review.

The characteristics of healthy controls, including age, gender, smoking and drinking, were collected via a questionnaire. Individuals who had smoked daily for more than 1 year were considered smokers, and those who consumed one or more alcoholic drinks per week for at least one year were considered drinkers. The protocol of this study was in accordance with the Declaration of Helsinkiand approved by the Institutional Review Board of the Nanjing First Hospital, and written informed consent was obtained from all the participants.

### DNA extraction and genotyping

The genotypes of all polymorphisms were detected with the SequenomMassARRAY platform, as previously described [10, 11]. First, DNA was extracted from whole-blood samples and concentrated by using GoldMag-Mini Whole Blood Genomic DNA Purification Kit according to the manufacture’s protocol (GoldMag Co. Ltd. Xi’an, China), and then DNA purity was measured by spectrometry (DU530 U*V*/VIS spectrophotometer, Beckman Instruments, Fullerton, CA, US). The qualified DNA samples were genotyped using the SequenomMassARRAY platform followed the standard protocol recommended by the manufacturer of a Sequenom Mass-ARRAY®RS1000(Sequenom, Inc.). Multiplexed SNP MassEXTENDED assay was designed by SequenomMassARRAY Assay Design 3.0 Software [12]. Finally, data management and analysis were performed using SequenomTyper 4.0 Software [12, 13].

### *H. pylori* infection detection

*H. pylori* infection status of enrolled participants has been determined by serology using a commercial *H. pylori* Immunogold Testing Kit (KangmeiTianhong Biotech (Beijing) Co., Ltd., Beijing, China), which has been validated in the Chinese population with sensitivity of 98.29% and specificity of 98.51% for the detection of *H. pylori* infection.

### Statistical analysis

The Hardy-Weinberg equilibrium in the healthy control group was tested by using a goodness of fit chi-square test. The statistical analysis for genotype distribution was performed by the χ2 test, and odds ratios (OR) and 95% confidence intervals (CIs) were calculated using logistic regression model. The dominant model, co-dominant model, and additive model were the test for all polymorphisms, with the dominant and co-dominant models being used only if the additive model is significant or there is a previous hypothesis to do this.

Survival curves were analyzed by the Kaplan-Meier method, and the Hazard ration (HR) and 95% CIs were calculated using Cox proportional hazards regression model. The *P* value < 0.05 was considered statistically significant difference. The haplotype analysis was performed using online software SHEsis (analysis.bio-x.cn/myAnalysis.php).

## Results

### Characteristics of the participants

There was no significant difference in age (cases: 65.55 ± 11.92 years, healthy controls: 64.85 ± 11.83; *p* = 0.694), gender (cases: male73.60%, healthy controls: male73.27%; *p* = 0.782), smoking (cases: 23.08%, healthy controls: 24.29%; *p* = 0.658), and drinking (cases: 11.02%, healthy controls: 9.59%; *p* = 0.465) between cases and controls. For *H. pylori* infection status, the ratio of *H. pylori* infection in cases (54.47%) was higher than that in healthy controls (49.18%), however there was no significant difference between the two groups (*p* = 0.099), as presented in Table 1.

**Table 1:** Clinical characteristics of the participants

Variables	Cases, n (%)	Controls, n (%)	*p*-Value
Total	481	490	
Age (mean ± SD)	65.55 ± 11.92	64.85 ± 11.83	0.694^a^
>60	168	167	0.782^b^
≤ 60	313	323	
Gender			
Male	354(73.60)	359(73.27)	0.907 ^b^
Female	127(26.40)	131(26.73)	
Drinking			
Yes	53(11.02)	47(9.59)	0.465 ^b^
No	428(88.98)	443(90.41)	
Smoking			
Yes	111(23.08)	119(24.29)	0.658 ^b^
No	370(76.92)	371(75.71)	
*Helicobacter pylori* infection status			
Positive	262(54.47)	241(49.18)	0.099 ^b^
Negative	219(45.53)	249(50.81)	
Differentiation			
Low	195(40.54)		
Med and high	286(59,46)		
Clinical stages			
T1-T2	159(33.06)		
T3-T4	322(66.94)		
Tumor Site			
GCA	140(29.11)		
NGCA	341(70.89)		

*GCA* gastric cardiac adenocarcinoma, *NGCA* non-gastric cardiac adenocarcinoma

^a^Independent *t*-test. ^b^Two-sided χ2 test for distributions between cases and controls

For the clinical pathological characteristics, a total of 195 (40.54%) and 286 (59.46%) patients had low and median to high pathological differentiation, respectively. For the tumor site classification, a total of 159 (33.06%) and 322 (66.94%) patients were classified to TNM stage T1-T2 and T3-T4, respectively. For the tumor location, a total of 140 (29.11%) and 341(70.89%) patients were diagnosed as gastric cardiac adenocarcinoma (GCA) and non-cardiatic GC (NCGC), respectively.

### Association between polymorphisms and risk of GC

The genotype distributions of the selected polymorphisms in cases and controls are presented in Table 2. The observed frequencies of all tested genotypes in controls did not deviate from Hardy-Weinberg equilibrium (HWE) (rs10074991: *p* = 0.129; rs13361707: *p* = 0.152; rs1044129: *p* = 0.368; rs154268: *p* = 0.140; rs6882903: *p* = 0.842; rs3805490: *p* = 0.929; rs461404: *p* = 0.155).

**Table 2:** Distribution of the genotypes in all participants

Genotype	Controls, n (%)	Patients, n (%)	OR (95% CI)^a^	*p*-Value
rs10074991				
AA	128(26.12)	104(21.62)	Reference	
AG	261(53.27)	255(53.01)	1.19(0.87,1.62)	0.283
GG	101(20.61)	122(25.36)	1.44(0.99,2.09)	0.056
AG/GG	362(73.88)	377(78.38)	1.26(0.94,1.70)	0.127
Additive model			1.21(1.01, 1.46)	0.042
rs13361707				
TT	129(26.33)	103(21.41)	Reference	
TC	260(53.06)	256(53.22)	1.22(0.89,1.66)	0.219
CC	101(20.61)	122(25.36)	1.47(1.01,2.14)	0.043
TC/CC	361(73.67)	365(75.88)	1.29(0.96,1.74)	0.093
Addictive model			1.22(1.02,1.47)	0.033
rs154268				
TT	297(60.61)	271(56.34)	Reference	
TC	176(35.92)	179(37.21)	1.13(0.86,1.47)	0.388
CC	17(3.47)	31(6.44)	1.96(1.06,3.63)	0.033
TC/CC	193(39.39)	210(43.66)	1.20(0.93,1.56)	0.158
Additive model			1.24(1.00,1.53)	0.053
rs6882903				
CC	342(69.80)	312(64.86)	Reference	
CA	134(27.35)	149(30.98)	0.86(0.41,1.80)	0.687
AA	14(2.86)	20(4.16)	1.56(0.77,3.15)	0.217
CA/AA	148(30.20)	169(35.14)	1.26(0.96,1.65)	0.097
Additive model			1.23(0.98,1.55)	0.078
rs3805490				
TT	279(56.94)	280(58.21)	Reference	
TA	181(36.94)	170(35.34)	0.93(0.71,1.21)	0.567
AA	30(6.12)	31(6.44)	1.02(0.60,1.73)	0.953
TA/AA	211(43.06)	201(41.79)	0.94(0.73,1.21)	0.627
Additive model			0.97(0.79,1.19)	0.756
rs461404				
AA	298(60.82)	270(56.13)	Reference	
GA	175(35.71)	179(37.21)	1.14(0.87,1.49)	0.341
GG	17(3.47)	32(6.65)	2.05(1.11,3.78)	0.022
GA/GG	192(39.18)	211(43.87)	1.22(0.95,1.58)	0.125
Additive model			1.26(1.01,1.56)	0.037

^a^Adjusted for age, gender, smoking, drinking, and *Helicobacter pylori* infection

Rs10074991 GG genotype had a borderline significantly increased risk of GC (adjusted OR = 1.44, 95%CI: 0.99–2.09, *p* = 0.056), and the additive model shows rs10074991 is an increased risk factor for GC (adjusted OR = 1.21, 95%CI: 1.01–1.46, *p* = 0.042). In similar, an increased risk of rs13361707 was also observed for GC (TC vs. GG: adjusted OR = 1.47, 95%CI: 1.01–2.14, *p* = 0.043; additive model: adjusted OR = 1.22, 95%CI: 1.02–1.47, *p* = 0.033). Besides, the results have also revealed that rs154268 and rs461404 are associated with increased GC risk (rs154268 TC: adjusted OR = 1.96, 95%CI: 1.06–3.63, *p* = 0.033; rs154268 additive model: adjusted OR = 1.24, 95%CI: 1.00–1.53, *p* = 0.053; rs461404 GA: adjusted OR = 2.05, 95%CI: 1.11–3.78, *p* = 0.022; rs461404 additive model: adjusted OR = 1.26, 95%CI: 1.01–1.56, *p* = 0.037). However, there was no significant association between rs6882903 and rs3805490 and risk of GC, as summarized in Table 2.

### Stratification analysis

To further assess the four potential susceptible polymorphisms (rs10074991, rs13361707, rs154268 and rs461404) to the risk of GC, a stratified analysis was performed by subgroups of participants’ clinical characteristics (age, gender, *H. pylori* infection status), and tumor pathological characteristics (tumor site, tumor differentiation, and clinical stage).

In China, men usually retire at age of 60, which means they retain a stable and sustainable life style (the environmental factors), so we choose 60 years as the cut-off value for the subgroup analysis. In the subgroup of age ≤ 60, rs10074991GG (adjusted OR = 1.93, 95%CI: 1.00–3.73, *p* = 0.050), rs13361707CC (adjusted OR = 2.00, 95%CI: 1.04–3.84, *p* = 0.039) and rs461404GG (adjusted OR = 3.12, 95%CI: 1.05–9.28, *p* = 0.040) were associated with increased GC risk. However, in the group of age**>**60, there was no significant association of these four polymorphisms with the risk of GC. For the subgroup of gender, in the male group, rs10074991 (additive model: adjusted OR = 1.25, 95%CI: 1.01–1.56, *p* = 0.046) and rs13361707 (CC: adjusted OR = 1.44, 95%CI: 1.01–2.06, *p* = 0.044; additive model: adjusted OR = 1.27, 95%CI: 1.02–1.58, *p* = 0.034) contributed to increased risk of GC. In similar, in the subgroup of positive *H. pylori* infection, a borderline significantly increased risk of rs10074991 (AG: adjusted OR = 1.68, 95%CI: 0.98–2.88, *p* = 0.060; additive model: adjusted OR = 1.30, 95%CI: 0.99–1.69, *p* = 0.057) and rs13361707 (TC: adjusted OR = 1.75, 95%CI: 1.02–3.00, *p* = 0.042; additive model: adjusted OR = 1.32, 95%CI: 1.01–1.73, *p* = 0.041) was observed for GC, as shown in Table 3. For the subgroup of pathological characteristics of tumor, the four polymorphisms were significant associated with increased risk of NCGC, but not GCA. Moreover, the significant associations of these four polymorphisms were observed in the subgroup of patients with tumor in median or high differentiation or T3-T4, but not for low differentiation or T1-T2, as shown in Table 4.

**Table 3:** *PRKAA1* Polymorphisms with Gastric Cancer Risk by Clinical Characteristics of Participants

Genotype	Age						Sex					*Helicobacter pylori* infection*.*						
	≤60			>60			Male		Female			Positive			Negative			
	Ca/Co	OR (95% CI)	P	Ca/Co	OR (95% CI)^a^	P	Ca/Co	OR (95% CI)^a^	P	Ca/Co	OR (95% CI)^a^	P	Ca/Co	OR (95% CI)^a^	P	Ca/Co	OR (95% CI)^a^	P
rs10074991																		
AA	33/45	Reference		71/83	Reference		69/62	Reference		35/36	Reference		52/57	Reference		52/71	Reference	
AG	90/90	1.31(0.76,2.25)	0.331	165/171	1.10(0.75,1.62)	0.630	197/193	1.35(0.93,1.95)	0.117	58/68	0.77(0.42,1.41)	0.399	142/139	1.12(0.72,1.75)	0.613	113/122	1.26(0.81,1.96)	0.302
GG	45/32	1.93(1.00,3.73)	0.050	77/69	1.20(0.76,1.91)	0.436	88/74	1.51(0.97,2.37)	0.070	34/27	1.27(0.63,2.58)	0.503	68/45	1.68(0.98,2.88)	0.060	54/56	1.31(0.77,2.22)	0.318
AG/GG	135/122	1.46(0.87,2.45)	0.154	242/240	1.15(0.80,1.66)	0.461	285/267	1.40(0.98,1.99)	0.067	92/95	0.91(0.52,1.59)	0.734	210/184	1.26(0.82,1.93)	0.292	167/178	1.27(0.84,1.94)	0.255
Additive model		1.40(1.01,1.93)	0.043		1.12(0.89,1.41)	0.332		1.25(1.01,1.56)	0.046		1.10(0.77,1.55)	0.611		1.30(0.99,1.69)	0.057		1.14(0.88,1.48)	0.313
rs13361707																		
TT	33/46	Reference		70/83	Reference		68/93	Reference		36/35	Reference		51/58	Reference		52/71	Reference	
TC	90/89	1.35(0.79,2.33)	0.273	166/171	1.12(0.76,1.65)	0.563	198/192	1.40(0.96,2.03)	0.080	58/68	0.77(0.42,1.41)	0.399	143/138	1.18(0.76,1.84)	0.465	113/122	1.26(0.81,1.96)	0.302
CC	45/32	2.00(1.04,3.84)	0.039	77/69	1.22(0.77,1.94)	0.402	88/74	1.56(1.00,2.44)	0.052	34/27	1.27(0.63,2.58)	0.503	68/45	1.75(1.02,3.00)	0.042	54/56	1.31(0.77,2.22)	0.318
TC/CC	135/121	1.51(0.90,2.53)	0.119	242/240	1.17(0.81,1.69)	0.408	286/266	1.44(1.01,2.06)	0.044	92/95	0.91(0.52,1.59)	0.734	211/183	1.32(0.86,2.03)	0.200	167/178	1.27(0.84,1.94)	0.255
Additive model		1.41(1.02,1.95)		0.036	1.13(0.90,1.42)	0.305		1.27(1.02,1.58)	0.034		1.10(0.77,1.55)	0.611		1.32(1.01,1.73)	0.041		1.14(0.88,1.48)	0.313
rs154268																		
TT	92/106	Reference		179/191	Reference		195/213	Reference		76/84	Reference		151/146	Reference		120/151	Reference	
TC	64/56	1.32(0.83,2.10)	0.241	115/120	1.03(0.74,1.44)	0.844	140/134	0.81(0.60,1.10)	0.357	39/42	0.95(0.55,1.65)	0.866	91/86	1.03(0.71,1.49)	0.891	88/90	1.21(0.83,1.78)	0.315
CC	12/5	2.77(0.92,8.33)	0.069	19/12	1.63(0.76,3.51)	0.210	19/12	1,73(0.81,3.67)	0.153	12/5	2.44(0.82,7.29)	0.111	20/9	2.16(0.95,4.91)	0.068	11/8	1.77(0.69,4.55)	0.238
TC/CC	76/61	1.45(0.92,2.26)	0.107	134/132	1.09(0.80,1.50)	0.581	159/146	1.20(0.89,1.62)	0.230	51/47	1.12(0.67,1.87)	0.668	111/95	1.13(0.79,1.62)	0.496	99/98	1.27(0.88,1.84)	0.208
Additive model		1.46(1.00,2.12)	0.048		1.13(0.87,1.48)	0.355		1.21(0.94,1.56)	0.142		1.23(0.82,1.85)	0.308		1.21(0.90,1.62)	0.201		1.26(0.92,1.73)	0.153
rs461404																		
AA	91/106	Reference		179/192	Reference		195/214	Reference		75/84	Reference		151/146	Reference		119/152	Reference	
GA	64/56	1.34(0.84,2.13)	0.218	115/119	1.05(0.75,1.45)	0.792	139/133	1.16(0.85,1.58)	0.347	40/42	1.00(0.58,1.72)	0.991	91/86	1.03(0.71,1.49)	0.891	88/89	1.25(0.85,1.83)	0.255
GG	13/5	3.12(1.05,9.27)	0.040	19/12	1.64(0.76,3.53)	0.207	20/12	1.84(0.87,3.87)	0.109	12/5	2.46(0.82,7.36)	0.108	20/9	2.16(0.95,4.91)	0.068	12/8	1.96(0.77,4.97)	0.156
GA/GG	77/61	1.49(0.95,2.32)	0.082	134/131	1.10(0.81,1.52)	0.537	159/145	1.21(0.90,1.64)	0.205	52/47	1.16(0.69,1.93)	0.573	111/95	1.13(0.79,1.62)	0.496	100/97	1.31(0.91,1.90)	0.150
Additive model		1.51(1.04,2.19)	0.030		1.14(0.88,1.49)	0.327		1.23(0.95,1.59)	0.113		1.26(0.84,1.89)	0.261		1.21(0.90,1.62)	0.201		1.31(0.95,1.79)	0.098

^a^Adjusted for age, gender, smoking, drinking, and *Helicobacter pylori* infection; Ca, case; Co, control

**Table 4:** *PRKAA1* Polymorphisms with Gastric Cancer Risk by Tumor Classification

Genotype	Co	Site						DIF						TNM					
		GCA			NGCA			Low			Med-high			T1-T2		T3-T4			
		Ca	OR (95% CI)^a^	P	Ca	OR (95% CI)^a^	P	Ca	OR (95% CI)^a^	P	Ca	OR (95% CI)^a^	P	Ca	OR (95% CI)^a^	P	Ca	OR (95% CI)^a^	P
rs10074991																			
AA	128	43	Reference		61	Reference		47	Reference		57	Reference		35	Reference		69	Reference	
AG	261	73	0.80(0.51,1.24)	0.312	182	1.45(1.01,2.09)	0.043	104	1.09(0.73,1.63)	0.683	151	1.28(0.88,1.86)	0.203	88	1.23(0.79,1.94)	0.362	167	1.16(0.82,1.66)	0.409
GG	101	24	0.66(0.37,1.16)	0.150	98	2.02(1.32,3.07)	0.001	44	1.17(0.71,1.93)	0.528	77	1.66(1.08,2.57)	0.022	36	1.33(0.77,2.29))	0.308	86	1.51(0.99,2.28)	0.054
AG/GG	362	97	0.77(0.50,1.16)	0.210	280	1.16(1.14,2.28)	0.007	148	1.20(0.76,1.65)	0.570	229	1.38(0.97,1.98)	0.074	124	1.27(0.82,1.95)	0.283	253	1.26(0.90,1.77)	0.171
Additive model			0.82(2.62,1.08)	0.163		1.43(1.16,1.76)	0.001		1.09(0.86,1.40)	0.475		1.31(1.05,1.62)	0.016		1.17(0.89,1.53)	0.255		1.24(1.01,1.53)	0.039
rs13361707																			
TT	129	43	Reference		60	Reference		47	Reference		56	Reference		35	Reference		68	Reference	
TC	260	73	0.80(0.52,1.25)	0.330	183	1.51(1.05,2.17)	0.027	104	1.10(0.73,1.65)	0.641	152	1.32(0.91,1.93)	0.141	88	1.25(0.80,1.97)	0.331	168	1.20(0.84,1.71)	0.320
CC	101	24	0.66(0.37,1.17)	0.157	98	2.08(1.36,3.16)	0.001	44	1.19(0.72,1.95)	0.495	78	1.71(1.11,2.65)	0.015	36	1.34(0.78,2.31)	0.290	86	1.55(1.02,2.34)	0.040
TC/CC	361	97	0.77(0.51,1.17)	0.225	281	1.67(1.18,2.36)	0.004	148	1.13(0.77,1.67)	0.529	230	1.42(1.00,2.05)	0.050	124	1.28(0.83,1.98)	0.257	254	1.30(0.93,1.82)	0.125
Additive model			0.82(0.62,1.09)	0.171		1.45(1.18,1.78)	0.001		1.10(0.86,1.40)	0.451		1.32(1.07,1.64)	0.012		1.18(0.90,1.54)	0.240		1.26(1.02,1.55)	0.030
rs154268																			
TT	297	88	Reference		183	Reference		115	Reference		156	Reference		90	Reference		181	Reference	
TC	176	48	0.89(0.59,1.33)	0.567	131	1.24(0.92,1.66)	0.153	70	1.05(0.74,1.49)	0.794	109	1.18(0.86,1.61)	0.299	63	1.12(0.85,1.82)	0.258	116	1.46(0.85,1.82)	0.704
CC	17	4	0.77(0.25.2.39)	0.657	27	2.54(1.34,4.81)	0.004	10	1.46(0.65,3.31)	0.364	21	2.38(1.20,4.58)	0.010	6	1.14(0.43,3.02)	0.790	25	2.46(1.28,4.70)	0.007
TC/CC	193	52	0.88(0.60,1.31)	0.535	158	1.36(1.03,1.81)	0.032	80	1.09(0.78,1.53)	0.614	130	1.28(0.95,1.73)	0.099	69	1.24(0.86,1.79)	0.253	141	1.19(0.89,1.58)	0.240
Additive model			0.89(0.63,1.26)	0.508		1.40(1.10,1.76)	0.005		1.12(0.84,1.49)	0.449		1.33(1.04,1.70)	0.025		1.19(0.87,1.63)	0.289		1.27(1.00,1.61)	0.046
rs461404																			
AA	298	88	Reference		182	Reference		114	Reference		156	Reference		90	Reference		180	Reference	
GA	175	48	0.90(0.60,1.34)	0.596	131	1.25(0.93,1.68)	0.132	71	1.08(0.76,1.54)	0.668	108	1.18(0.86,1.61)	0.383	63	1.25(0.86,1.83)	0.243	116	1.08(0.80,1.46)	0.628
GG	17	4	0.78(0.25,2.40)	0.659	28	2.67(1.42,5.04)	0.002	10	1.48(0.65,3.34)	0.349	22	2.47(1.27,4.79)	0.007	6	1.23(0.58,2.61)	0.787	26	2.59(1.36,4.93)	0.004
GA/GG	192	52	0.89(0.60,1.32)	0.563	159	1.38(1.05,1.84)	0.023	81	1.12(0.80,1.58)	0.509	130	1.29(0.96,1.74)	0.116	69	1.25(0.86,1.80)	0.240	142	1.21(0.91,1.62)	0.187
Additive model			0.90(0.63,1.27)	0.532		1.42(1.13,1.79)	0.003		1.14(0.85,1.52)	0.373		1.34(1.05,1.72)	0.019		1.19(0.87,1.64)	0.276		1.30(1.03,1.65)	0.029

^a^Adjusted for age, gender, smoking, drinking, and *Helicobacter pylori* infection; *GCA* gastric cardia adenocarcinoma, *NGCA* non-gastric cardia adenocarcinoma, *Ca*, case *Co* control

### Haplotype analysis of polymorphisms in *PRKAA1*

**Fig. 1:**
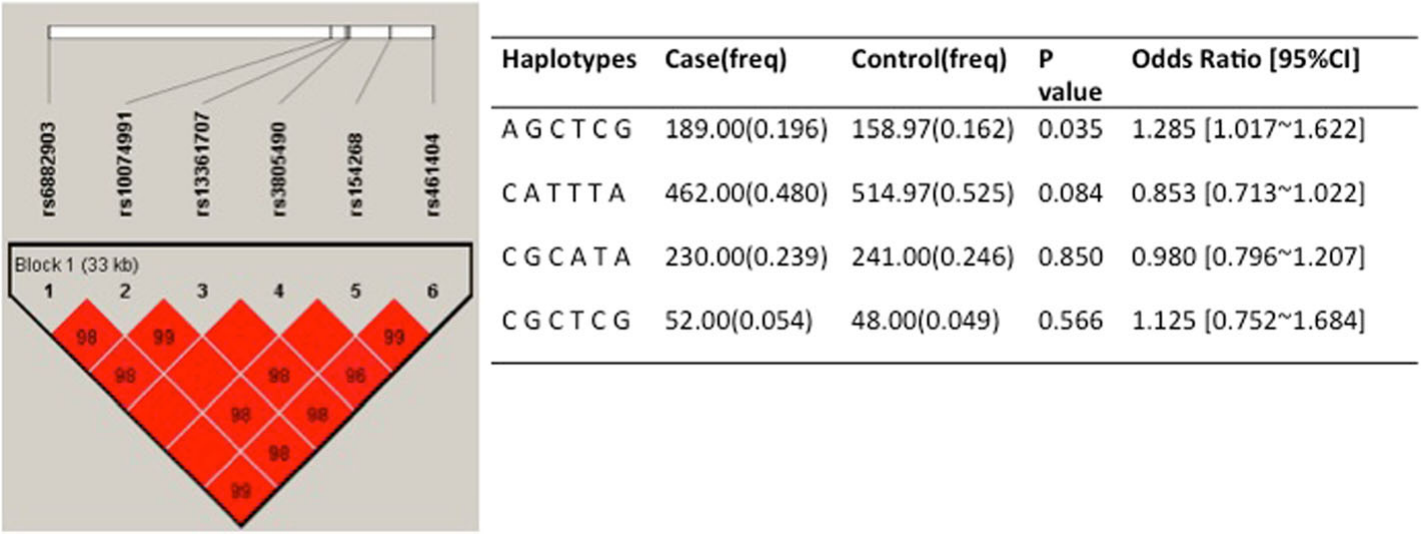
Haplotype analysis of polymorphisms indicating the susceptibility to gastric cancer risk. The linkage disequilibrium (LD) map according to the genotype data, the color and figure show the linkage disequilibrium coefficient with D’ values The prevalence of haplotype A-G-C-T-C-G (rs6882903, rs10074991, rs13361707, rs3805490, rs154268, rs461404) was significantly higher among cases (19.6%) compared to controls (16.2%) (haplotype-specific *p* = 0.035), and those with this haplotype have 1.29 times higher risk of gastric cancer (OR = 1.29, 95%CI: 1.02–1.62, *p* = 0.035) compared to noncarriers

The enrolled six polymorphisms locate in the intron or upstream of *PRKAA1*, so these sites may be in linkage disequilibrium with each other. Therefore, the combined susceptibility of these six polymorphisms to GC risk was calculated by haplotype analysis. The results indicated that the haplotype A-G-C-T-C-G (rs6882903, rs10074991, rs13361707, rs3805490, rs154268, rs461404) is associated with the increased risk of GC (OR = 1.29, 95%CI: 1.02–1.62, *p* = 0.035), as compared with other haplotypes (Fig. 1).

### Association between polymorphisms and clinical outcome of patients

A total 481 patients were followed up for the survival state. The association of polymorphisms with the overall survival (OS) of patients was assessed for their predictive value for patients with heterozygous and homozygous genotype, or their combination, compared to the wild genotype. The results revealed that rs10074991 (AG: adjusted HR = 1.80, 95%CI:1.21–2.67, *p* = 0.004; GG: adjusted HR = 1.75, 95%CI: 1.13–2.70, *p* = 0.012; AG/GG: HR = 1.78, 95%CI: 1.21–2.61, *p* = 0.003) and rs13361707 (TC: adjusted HR = 1.85, 95%CI: 1.24–2.77, *p* = 0.003; CC: adjusted HR = 1.79, 95%CI: 1.16–2.78, *p* = 0.009; TC/CC: adjusted HR = 1.83, 95%CI: 1.24–2.70, *p* = 0.002) were associated with poor OS of patients with NCGC, indicating these two polymorphisms have a significant prediction value for the patients with NCGC, as shown in Table 5.

**Table 5:** *PRKAA1* Polymorphisms with clinical outcome of patients with NGCA

Genotype	All patients		NGCA					
	HR (95% CI)	*p*-Value	HR (95% CI)	*p*-Value	HR (95% CI)^a^	*p*-Value	HR (95% CI)^b^	*p*-Value
rs10074991								
AA	Reference		Reference		Reference		Reference	
AG	1.16(0.87,1.55)	0.300	1.62(1.10,2.38)	0.015	1.63(1.10,2.41)	0.015	1.80(1.21,2.67)	0.004
GG	1.15(0.83,1.59)	0.401	1.64(1.08,2.49)	0.020	1.71(1.12,2.60)	0.012	1.75(1.13,2.70)	0.012
AG/GG	1.16(0.88,1.52)	0.290	1.62(1.12,2.35)	0.011	1.66(1.14,2.41)	0.008	1.78(1.21,2.61)	0.003
rs13361707								
TT	Reference		Reference		Reference		Reference	
TC	1.18(0.89,1.57)	0.253	1.67(1.13,2.47)	0.010	1.68(1.13,2.50)	0.010	1.85(1.24,2.77)	0.003
CC	1.16(0.84,1.61)	0.364	1.68(1.10,2.56)	0.016	1.76(1.15,2.68)	0.009	1.79(1.16,2.78)	0.009
TC/CC	1.18(0.90,1.54)	0.246	1.67(1.15,2.44)	0.007	1.71(1.17,2.50)	0.006	1.83(1.24,2.70)	0.002
rs154268								
TT	Reference		Reference					
TC	1.08(0.85,1.36)	0.525	1.18(0.90,1.56)	0.242				
CC	1.23(0.78,1.94)	0.367	1.29(0.78,2.12)	0.322				
TC/CC	1.10(0.88,1.17)	0.405	1.20(0.92,1.56)	0.183				
rs6882903								
CC	Reference		Reference					
CA	0.97(0.76,1.24)	0.796	1.12(0.84,1.48)	0.443				
AA	1.48(0.89,2.47)	0.130	1.56(0.90,2.72)	0.113				
CA/AA	1.02(0.81,1.29)	0.869	1.17(0.89,1.53)	0.252				
rs3805490								
TT	Reference		Reference					
TA	1.03(0.82,1.30)	0.807	1.07(0.81,1.41)	0.626				
AA	0.82(0.51,1.31)	0.404	0.97(0.55,1.68)	0.898				
TA/AA	0.99(0.80,1.24)	0.953	1.06(0.81,1.37)	0.691				
rs461404								
AA	Reference		Reference					
GA	1.08(0.86,1.37)	0.506	1.19(0.90,1.56)	0.352				
GG	1.25(0.80,1.94)	0.333	1.31(0.80,2.13)	0.228				
GA/GG	1.11(0.89,1.38)	0.379	1.21(0.93,1.57)	0.165				

## Discussion

This study revealed that *PRKAA1* genetic polymorphismsrs13361707CC, rs10074991GG, rs461404GG, and rs154268CC were associated with increased risk of GC. The susceptibility of these four polymorphisms to the risk of GC were here observed in the subgroup of age ≤ 60, male, NCGC, median to high differentiation and T3-T4 subgroup. Polymorphisms rs13361707 and rs10074991 were associated with poor survival of patients with NCGC.

Variant rs13361707 is located in the first intron of *PRKAA1* at 5p13.1, which was primarily found to be associated with NCGC risk by a GWAS in a Chinese population(1006 non-cardia gastric cancer and 2273 controls, and confirmed with 3288 with non-cardia gastric cancer and 3609 controls) [8], and the significant association was duplicated by other studies on Chinese population(1124 cases and 1,194controls) [14] and on Korean population (Kim et al.: 477 case-control pairs; Song et al.: 3245 cases and 1700 controls) [15, 16]. This study observed that rs13361707 CC genotype was associated with increased risk of GC, and C allele carriers had a higher risk of NCGC, but not of GCA, indicating the association of rs13361707with the increased GC risk is specific to NCGC. Etiological studies have found differences between GCA and NCGC, concerning e.g. *H. pylori* infection [17, 18], or body mass index [19], and which was confirmed by epidemiological study that also suggested the susceptibility of genetic polymorphism to GC is different for NCGC and GCA [20]. Moreover, in the subgroup of positive *H. pylori* infection, our study showed rs13361707CC genotype is associated with increased risk of GC, indicating the interaction of rs13361707 and *H. pylori* can enhance the GC risk, which is consistent with the results of previous study [21]. The polymorphism rs13361707 is located in the first intron of *PRKAA1* gene, which is a cellular energy sensor maintaining energy homeostasis, and contributes to cancer development by regulating mRNA translation and protein synthesis [22, 23]. Although the function of rs13361707 is largely unknown, several published studies and the current work indicated that risk of rs13361707 for GC was associated with the type of GC, and its susceptibility may be influenced by *H. pylori* infection [5].

This study also showed that rs10074991GG genotype is borderline significantly associated with increased risk of GC, and stratification analysis revealed the genotype to be associated with increased risk of NCGC, which is consistent with the reports of Hu et al. [20] that rs10074991 G allele linked with rs13361707 C allele (these two polymorphisms locate in the intron of *PRKAA1* with the distance of 1333 bp) was a risk factor of NCGC. Moreover, such an association was also reported by Kim et al. [15] in a Korean population [15]. However, the function of these two sites remains unclear and the mechanism has yet to be established.

In this study, rs154268 CC genotype was also found to be associated with increased risk of GC for all participants and especially for the subgroup of NCGC, tumor with median to high differentiation, and T3-T4, suggesting rs154268 could be associated with pathological characteristics of GC. Consistent with this, the rs154268 TC genotype was also previously reported to be associated with the risk of GC [15], indicating that the C allele is a risk factor for GC. Actually, this study revealed the linkage disequilibrium (LD) between rs154268 and rs461404 (D′ = 1.0), which means the result of rs461404 is in accord with that of rs154268. However, to date, there is no functional study regarding the potential functional role of these two polymorphisms in carcinogenesis. In general, in this study, the result of rs461404 was inconsistent with that of rs154268.

The present work showed that rs10074991 G and rs13361707 C allele carriers with NCGC have poor OS, and this association was still observed after being adjusted by basic clinical characteristics (age, gender, *H. pylori* infection, drinking, and smoking) or pathological characteristics (tumor differentiation, tumor stage), indicating these two polymorphisms were independent factors for predicting the clinical outcome for NCGC. To our knowledge, this is the first report to discuss the role of these two polymorphisms in prognosis for patients with NCGC, which however should be verified by a further research with larger samples.

There are some limitations of this study. First, the sample size is relatively small, which may limit the statistical power, especially for the multiple stratified analyses. Second, the polymorphisms discussed in this study were limited in number and based on previous knowledge of potential functional significance of polymorphisms that have been found to be related to GC risk. Thus, a more comprehensive tagging SNP-based approach and a haplotype block analysis would better assesses the association and provides more complete information regarding the associations of AMPK pathway genes and GC risk.

## Conclusions

This case-control study provided the evidence that rs13361707CC, rs10074991GG, rs461404GG, and rs154268CC are associated with increased GC risk, especially for NCGC, and that rs10074991 G and rs13361707 C alleles are independent prognostic factors for NCGC.

## Additional file

Additional file 1: Polymorphism position and minor allele frequency. (DOC 35 kb)

## Abbreviations

AMPK: 5’-AMP-activated protein kinase
CI: confidence intervals
GCA: gastric cardiac adenocarcinoma
GWAS: genome-wide association study
*H. pylori*: *Helicobacter pylori*
HR: hazard ration
HWE: Hardy-Weinberg Equilibrium
NCGC: non-cardiac gastric cancer
OR: odds ratios
OS: overall survival

## References

[1] Torre LA, Bray F, Siegel RL, Ferlay J, Lortet-Tieulent J, Jemal A (2015). Global cancer statistics, 2012. CA Cancer J Clin.

[2] Ang TL, Fock KM (2014). Clinical epidemiology of gastric cancer. Singap Med J.

[3] Karimi P, Islami F, Anandasabapathy S, Freedman ND, Kamangar F (2014). Gastric cancer: descriptive epidemiology, risk factors, screening, and prevention. Cancer Epidemiol Biomarkers Prev.

[4] Hemminki K, Sundquist J, Ji J (2007). Familial risk for gastric carcinoma: an updated study from Sweden. Br J Cancer.

[5] Mocellin S, Verdi D, Pooley KA, Nitti D (2015). Genetic variation and gastric cancer risk: a field synopsis and meta-analysis. Gut.

[6] Piao Y, Li Y, Xu Q, Liu JW, Xing CZ, Xie XD, Yuan Y (2015). Association of MTOR and AKT gene polymorphisms with susceptibility and survival of gastric Cancer. PLoS One.

[7] Hyland PL, Lin SW, Hu N, Zhang H, Wang L, Su H, Wang C, Ding T, Tang ZZ, Fan JH (2014). Genetic variants in fas signaling pathway genes and risk of gastric cancer. Int J Cancer.

[8] Shi Y, Hu Z, Wu C, Dai J, Li H, Dong J, Wang M, Miao X, Zhou Y, Lu F (2011). A genome-wide association study identifies new susceptibility loci for non-cardia gastric cancer at 3q13.31 and 5p13.1. Nat Genet.

[9] Dong Y, Chen J, Chen Z, Tian C, Lu H, Ruan J, Yang W (2015). Evaluating the Association of Eight Polymorphisms with Cancer susceptibility in a Han Chinese population. PLoS One.

[10] He B, Pan Y, Xu Y, Deng Q, Sun H, Gao T, Wang S (2015). Associations of polymorphisms in microRNAs with female breast cancer risk in Chinese population. Tumour Biol.

[11] He BS, Pan YQ, Lin K, Ying HQ, Wang F, Deng QW, Sun HL, Gao TY, Wang SK (2015). Evaluation the susceptibility of five polymorphisms in microRNA-binding sites to female breast cancer risk in Chinese population. Gene.

[12] Gabriel S, Ziaugra L, Tabbaa D: SNP genotyping using the Sequenom MassARRAY iPLEX platform. Curr Protoc Hum Genet. 2009;Chapter 2:Unit 2 12. 10.1002/0471142905.hg0212s60 19170031

[13] Thomas RK, Baker AC, Debiasi RM, Winckler W, Laframboise T, Lin WM, Wang M, Feng W, Zander T, MacConaill L (2007). High-throughput oncogene mutation profiling in human cancer. Nat Genet.

[14] Qiu LX, He J, Cheng L, Zhou F, Wang MY, Sun MH, Zhou XY, Li J, Guo WJ, Wang YN (2015). Genetic variant of PRKAA1 and gastric cancer risk in an eastern Chinese population. Oncotarget.

[15] Kim YD, Yim DH, Eom SY, Moon SI, Yun HY, Song YJ, Youn SJ, Hyun T, Park JS, Kim BS (2014). Risk of gastric cancer is associated with PRKAA1 gene polymorphisms in Koreans. World J Gastroenterol.

[16] Song HR, Kim HN, Kweon SS, Choi JS, Shim HJ, Cho SH, Chung IJ, Park YK, Kim SH, Choi YD (2013). Genetic variations in the PRKAA1 and ZBTB20 genes and gastric cancer susceptibility in a Korean population. Mol Carcinog.

[17] Kamangar F, Dawsey SM, Blaser MJ, Perez-Perez GI, Pietinen P, Newschaffer CJ, Abnet CC, Albanes D, Virtamo J, Taylor PR (2006). Opposing risks of gastric cardia and noncardia gastric adenocarcinomas associated with helicobacter pylori seropositivity. J Natl Cancer Inst.

[18] Kamangar F, Qiao YL, Blaser MJ, Sun XD, Katki H, Fan JH, Perez-Perez GI, Abnet CC, Zhao P, Mark SD (2007). Helicobacter pylori and oesophageal and gastric cancers in a prospective study in China. Br J Cancer.

[19] Abnet Christian C., Freedman Neal D., Hollenbeck Albert R., Fraumeni Joseph F., Leitzmann Michael, Schatzkin Arthur (2008). A prospective study of BMI and risk of oesophageal and gastric adenocarcinoma. European Journal of Cancer.

[20] Hu N, Wang Z, Song X, Wei L, Kim BS, Freedman ND, Baek J, Burdette L, Chang J, Chung C, et al. Genome-wide association study of gastric adenocarcinoma in Asia: a comparison of associations between cardia and non-cardia tumours. Gut. 2015. 10.1136/gutjnl-2015-309340 PMC5568652 26129866

[21] Cai M, Dai S, Chen W, Xia C, Lu L, Dai S, Qi J, Wang M, Wang M, Zhou L (2017). Environmental factors, seven GWAS-identified susceptibility loci, and risk of gastric cancer and its precursors in a Chinese population. Cancer Med.

[22] Krishan S, Richardson DR, Sahni S (2014). Gene of the month. AMP kinase (PRKAA1). J Clin Pathol.

[23] van Veelen W, Korsse SE, van de Laar L, Peppelenbosch MP (2011). The long and winding road to rational treatment of cancer associated with LKB1/AMPK/TSC/mTORC1 signaling. Oncogene.

