# Supplementary material for: Genetic variations in PRKAA1 predict the risk and progression of gastric Cancer
Source: BMC Cancer. 2018 Sep 25;18:923. doi: 10.1186/s12885-018-4818-3 (PMC6156979; doi:10.1186/s12885-018-4818-3)
Supplement: Supplementary file 1 — Polymorphism position and minor allele frequency. (DOC 35 kb) [file 12885_2018_4818_MOESM1_ESM.doc]

| rs number | Chromesome position | Allele | Global MAFs | MAFs of this study |
| --- | --- | --- | --- | --- |
| rs6882903 | 5:40765760 | A/C | A=0.4505 | A=0.1653 |
| rs10074991 | 5:40790449 | A/G | A=0.3656 | A=0.5276 |
| rs13361707 | 5:40791782 | C/T | T=0.3634 | T=0.5286 |
| rs3805490 | 5:40792051 | A/T | A=0.2288 | A=0.2459 |
| rs154268 | 5:40795766 | C/T | C=0.2794 | C=0.2143 |
| rs461404 | 5:40799438 | G/A | G=0.2674 | G=0.2133 |

Additional file 1 Polymorphism position and minor allele frequency.
